# Principal components analysis - K-means transposon element based foxtail millet core collection selection method

**DOI:** 10.1186/s12863-016-0343-z

**Published:** 2016-02-16

**Authors:** Ernesto Borrayo, Ryoko Machida-Hirano, Masaru Takeya, Makoto Kawase, Kazuo Watanabe

**Affiliations:** Gene Research Center, University of Tsukuba, 1-1-1 Tennodai, Tsukuba City, 305-8571 Ibaraki Japan; Genetc Resources Center, National Institute of Agrobiological Sciences, 2-1-2 Kannodai, Tsukuba City, 305-8602 Ibaraki Japan

**Keywords:** Core collection, Foxtail millet, K-means, Principal component analysis

## Abstract

**Background:**

Core collections are important tools in genetic resources research and administration. At present, most core collection selection criteria are based on one of the following item characteristics: passport data, genetic markers, or morphological traits, which may lead to inadequate representations of variability in the complete collection. The development of a comprehensive methodology that includes as much element data as possible has been explored poorly. Using a collection of (*Setaria italica* sbsp. *italica* (L.) P. Beauv.) as a model, we developed a method for core collection construction based on genotype data and numerical representations of agromorphological traits, thereby improving the selection process.

**Results:**

Principal component analysis allows the selection of the most informative discriminators among the various elements evaluated, regardless of whether they are genetic or morphological, thereby providing an adequate criterion for further K-mean clustering. Overall, the core collections of *S. italica* constructed using only genotype data demonstrated overall better validation scores than other core collections that we generated. However, core collection based on both genotype and agromorphological characteristics represented the overall diversity adequately.

**Conclusions:**

The inclusion of both genotype and agromorphological characteristics as a comprehensive dataset in this methodology ensures that agricultural traits are considered in the core collection construction. This approach will be beneficial for genetic resources management and research activities for *S. italica* as well as other genetic resources.

**Electronic supplementary material:**

The online version of this article (doi:10.1186/s12863-016-0343-z) contains supplementary material, which is available to authorized users.

## Background

The exploitation of genetic resources has been a primary concern for several governmental and nongovernmental agricultural institutions around the world [1], where the interest may vary from economically exploitable variant crops [2], to sociocultural [3], health-related [4], and biological-related studies (phylogenetic relationships, phenotype-genotype relationships, and physiological-environmental behaviors [1]). However, most researchers must address the problem of data mining to obtain collections of an appropriate size [5].

Due to the size of some collections, complete collection (MC) data mining may sometimes be too expensive (both operative and monetary); therefore, core collections (CC) [6] and mini-core collections have emerged in recent decades [7].

Methods for obtaining an optimal CC have been explored widely [8–11], and several algorithms and informatics tools have been developed [12–15], but CCs still have many different objectives and various evaluation criteria [10].

Most CC-related studies are based on one or more of three principal characteristics: a) passport data, b) genotypic analysis, and c) morphological traits ([16]). As new genetic information becomes available, CC selection has increasingly used genotypic analysis as a good criterion, but the efficiency of specific molecular markers needs to be demonstrated for phenotypic traits of interest because both types of data are fundamental requirements of genetic breeding programs [17]. Several studies have utilized molecular markers in different collections, including the development of CCs based on widely used simple sequence repeats [11, 17, 18] and restriction fragment length polymorphisms [19], which have demonstrated the great potential of using genetic data for CC selection.

Foxtail millet (*Setaria italica* subsp. *italica* (L.) P. Beauv.) is one of the oldest cereals consumed by people in Eurasia, America, Africa, and Australia. Foxtail millet has a relatively small genome size (515 M) and it is has been adopted as a model organism [20, 21] because of its potential use in studies that involve grass species evolution, C_3_ and C_4_ photosynthesis, stress biology and biofuel [22–24].

Three recently active transposons (TE) have proved to be suitable genome-wide markers for evolutionary studies of *S. italica* [25]. We hypothesize that these markers may also be useful for CC selection in this species.

In this study, we combined principal components analysis (PCA) and the K-means method for CC selection [18] based on evaluations of traditional and newly described CC evaluation parameters [10]. This methodology allowed to include both genotypic and agromorphological traits (AT) in CC selection. Thus, we present a proof of concept for the potential use of TE and AT combined as selection criteria for CC construction in *S. italica*.

## Methods

### Core collection selection

#### Dataset used

The accessions used in this study originated from 38 different countries, which encompassed the major traditional geographical distribution (Asia, Eurasia, and Africa) of the study species. In order to obtain genomic information, transposon display (TD), a modified form of amplified fragment length polymorphism (AFLP) [26], was performed with some modifications using three TEs: *TSI-1* [tourist miniature interspersed nuclear elements (Tourist MITE)], *TSI-7* [long terminal repeats (LTR) retrotransposons], and *TSI-10* [short interpersed nuclear elements(SINE)], with different classes and characteristics [27]. These TEs were identified in the mutant alleles of *Waxy* (*GBSS1*), which controls the amylose content in the starch endosperm [27]. The genomic dataset obtained (*data 0*) comprised a total of 423 *S. italica* accessions, which were genotyped by TD [25]. AT data was downloaded and categorized from the National Institute of Agrobiological Sciences (NIAS) http://www.gene.affrc.go.jp/databases-plant_search_char_en.php?type=9 for 141 of the original 423 accessions. Eight ATs were categorized and mapped to binary data, which were represented as 28 “*m*” characteristics (*data II*) for discrete variables, and any possible phenotypic traits were treated as present/absent. Continuous variables were categorized arbitrarily into three groups and then treated as discrete variables using the same present/absent criteria. The original phenotypic values and their numerical representations are summarized in Additional file 1 (Online Resource 1). To facilitate comparisons of *data II* behavior, we created *data I*, which comprised the same 141 accessions used in *data II*, but with the genotypic information for *data 0*. In order to determine the feasibility of analyzing phenotypic traits with genotypic markers in a single step, we merged the *data I* and *data II* sets to obtain (*data III*), where each *m* element was treated as equal regardless of its TD or AT origin.

#### Principal component analysis - K-means analysis

Because the informativeness is different for each *m* element of *data*, PCA was performed in order to rearrange *data* into a new matrix. This procedure decreases the informativeness of subsequent elements and it discards elements with a variance that is equal to 0. This process generated two new matrices: one containing the original *m* characteristics mapped vectors (*x*) and the rearranged variance value matrix (*X*). Thus, matrix *X* contained *n* samples, which were formed of a numerical vector with *m*=*m*-(non-informative *m*). *m* can also be determined arbitrarily in order to work with only the most informative elements of *data*. To select the CCs, we performed PCA to arrange the data from the most significant to the least significant elements in terms of the difference information discriminator, but without affecting the element associations [28]. After rearranging the data, the score that represented each value was subjected to K-means clustering according to [29], which is an implementation that enhances the K-means algorithm in order to avoid empty clusters. For each K cluster, the sample with the lowest Euclidean distance from the cluster centromere was selected as a representative. The newly generated CC was evaluated according to several validation parameters, which have been used widely [8, 9] and reviewed in recent studies [10].

### Evaluation of the selected core collections

The selected CCs were analyzed based on their distribution according to a phylogenetic reconstruction. A genetic distance matrix and a neighbor-joining dendrogram were obtained using AFLP-SURV 1.0 [30] and the Phylogeny Inference Package (PHYLIP) 3.69 [31], respectively, for the 141 accessions present in *data I*. The *data I* dendrogram and the visualization of the CCs were obtained using MEGA 5.2 [32]. The geographical distributions of the CCs were digitalized and visualized using DIVA GIS http://www.diva-gis.org/.

According to [10], the best method for evaluating a CC depends on the purpose of the CC and ideally different datasets should be used in the evaluation, although it can be performed with the same data. Thus, they established three criteria based on the CC data dispersion: a) average distance between each MC sample and the nearest CC sample (ANE), b) average distance between each CC sample and the nearest CC sample (ENE), and c) average distance between CC samples (E), which are calculated as:

(1) $$ ANE_{tot}=\frac{1}{L}\sum\limits^{K}_{k=1}\sum\limits^{J}_{j=1}D(k-cMC_{j}),  $$

where *K* is the total of CC elements, *k* is each CC element, and *D* is the alignment-free genomic distance (GAFD) [33] between *k* and each *jthcMC* element, for which the closest CC element is *k*, including itself, thereby yielding *L* comparisons in total.

(2) $$ ENE_{tot}=\frac{1}{L}\sum\limits^{K}_{k=1}D(k-cCC),  $$

where *K* is the total of CC elements, *k* is each CC element, and *D* is the GAFD distance between *k* and its closest CC element *cCC*, excluding itself, thereby yielding *L* comparisons in total.

(3) $$ E_{tot}=\frac{1}{L}\sum\limits^{K}_{k=1}\sum\limits^{J}_{j=1}D(k-cCC_{j}),  $$

where *K* is the total of CC elements, *k* is each CC element, and *D* is the GAFD distance between *k* and all other *jth* CC elements, *cCC*, excluding itself, thereby yielding *L* comparisons in total.

The ideal value for ANE is 0, where each sample of CC represents itself and others exactly like it. It is useful to evaluate CCs where the objective is a homogeneous representation of the diversity in the MC. In addition, ENE and E are used to evaluate the data dispersion for the CC, where higher values indicate the better representation of extreme values.

Evaluation criteria based on statistical parameter comparisons between the CC and the MC are used mainly to determine whether the CC adequately represents the identity of the MC as well as its distribution. Widely used evaluation parameters that meet these criteria were applied as follows.

A homogeneity test was performed on each trait for CC and MC based on the means and variances. For each comparison, a global value was represented as the percentage of traits that were statistically different (*α*=0.05) according to a *t*−*test* for means (MD) and the *F*−*test* for variances (VD) [8].

The coincidence rate (CR) and variable rate (VR) were used to evaluate the properties of the CCs in terms of the MC, which are defined by:

(4) $$ CR=\frac{1}{M}\sum\limits^{M}_{m=1}\frac{R_{CC}}{R_{MC}}*100  $$

and

(5) $$ VR=\frac{1}{M}\sum\limits^{M}_{m=1}\frac{CV_{CC}}{CV_{MC}}*100,  $$

respectively, where *R* is the range and *CV* is the coefficient of variation for each *m* trait in the CC and MC, and *M* is the number of traits. According to ([9]), a valid CC has *CR*>80 and *MD*<20, which are the limits for the ideal representation of the MC identity and distribution. The coverage of alleles (CA) in a CC measures the percentage of alleles from the MC that are present in the CC, which is given by:

(6) $$ CA=\left[|1-(|1-ACC|/AMC)|\right]* \,100,  $$

where ACC is the set of alleles in the CC and AMC is the set of alleles present in the MC [12].

Excluding the phylogenetic reconstruction and geographical distribution, all of the methodological procedures were performed using FREEMAT v4.2 www.freemat.sourceforge.net.

The FREEMAT codes are available in Additional file 2 (Online Resource 2).

## Results and discussion

### Usefulness of transposon display markers for CC selection

Locus-specific molecular marker systems, such as SNPs [21, 34], microsatellites [35] and other indel events [34] are available for foxtail millet. These markers may provide useful information for CC selection, but the full coverage of the complete genome with these markers has some conceptual and methodological limitations. SNPs and indels provide relatively less information per locus due to their bi-allelic nature and over 10,000 SNPs may be required to discriminate a closely related populations [36]. Microsatellites may overcome these limitations, but testing microsatellites that cover the complete genome distribution also incur high laboratory expenses and time-consuming procedures [1].

The use of TEs as an alternative to locus-specific molecular marker systems is based on the assumption that a significant fraction of plant genomes comprise TEs [37], i.e., recently active display higher polymorphisms [38]. A considerably large number of alleles can be detected using TEs as genetic markers with a small number of primer sets. CC selection using TEs combined with the recently released foxtail millet genome sequence [21] will considerably increase the number of polymorphic markers. Thus, we proposed a method that does not require genomic information, or a large number of locus-specific genetic markers, which is based on an AFLP-like technique that could easily be transferred to other biological systems. This method will enhance the reliability of CC selection considerably, thereby refining the exploitation of genetic resources.

To demonstrate the efficiency of ATs and TEs as CC selection criteria, we used K-means as a practical approach to clustering based on Kai et al. [11], who stated that the use of the principal coordinates instead of raw data (i.e., microsatellite genotype data) before K-means clustering makes the clustering step less sensitive to changes in the noisiness of the raw data. We agree that dimensionality reduction can enhance clustering process and it is possible to reduce the number of dimensions analyzed during this methodological step. However, to avoid more variables in the ATs and TEs evaluation, we used all of the informative data and we will explore the significance of dimension reduction in future implementations.

### Validation of the CCs selected by different datasets

**Fig. 1:**
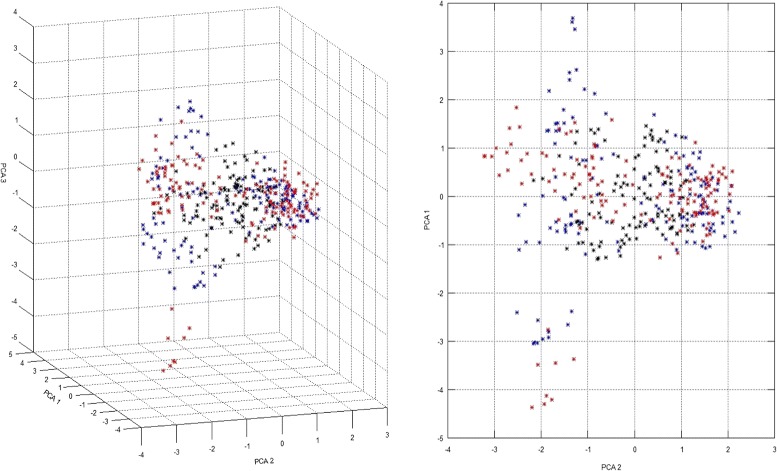
Principal component distributions for *data I* (*blue*), *data II* (*black*), and *data III* (*red*) in the first three (*left*) and two (*right*) principal components, respectively

**Fig. 2:**
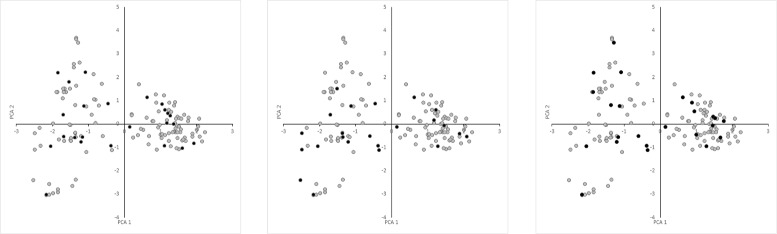
Principal component distributions of *data I* (*left*), *data II* (*center*), and *data III* (*right*) in *data I* for the first two principal components

The validation scores (VS) for different *K* values are presented in Table 1. As expected, the scores obtained by the CCs improved as their *K* values increased, which strongly suggests that the VSs are consistent with those reported previously [9, 10]. Interestingly, the VSs agreed with the *data I*, *data II*, and *data III* distributions (Fig. 1). When the CCs were constructed and evaluated using the same data (Figs. 2*(left)*, 3*(center)* and 4*(right)*), *data II* obtained better ANE and ENE results because these values should be affected considerably by the relationship between the data distribution and K value. This effect was supported when the CCs were constructed and evaluated using different data (Figs. 2*(center*&*right)*, 3*(left & right)* and 4*(left & center)*). Thus, the CCs constructed using *data I* and evaluated with *data II* obtained better results in terms of most of the VSs, but not vice versa. Initially, this may suggest that genotypic data are better for CC construction, but a genotype-based CC cannot ensure the inclusion of interesting agricultural traits. In general, the *data III* VS values were as expected between *data I* and *data II*, but there were some interesting exceptions. When they were compared using the same data, the ANE and ENE values with *data III* were lower than those obtained with the other datasets. This may be explained by the data distribution pattern (Figs. 2*(left)*, 3*(center)* and 4*(right)*). The data distribution of *data III* was wider, which would lead to poorer ANE values with the same *k* than when the data distribution is more compact. The same distribution effect obtained the opposite result when compared with different data, where in some cases *data III* obtained even better ANE values than *data I* and *data II*. The ENE values were also affected by the data distribution because wider distributions generated extreme value representations, which were more difficult to handle under the *k-mere* representations implemented in this study (i.e., the closest element to the centromere). A better ENE score may be obtained using different selection criteria, which will be addressed in future implementations of this concept.

**Fig. 3:**
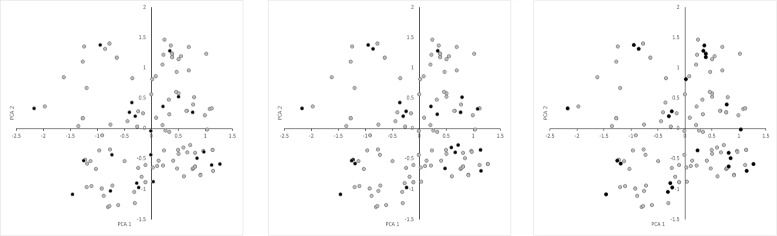
Principal component distributions of *data I* (*left*), *data II* (*center*), and *data III* (*right*) in *data II* for the first two principal components

**Fig. 4:**
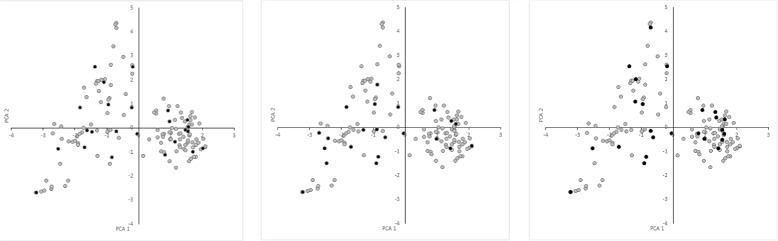
Principal component distributions of *data I* (*left*), *data II* (*center*), and *data III* (*right*) in *data III* for the first two principal components

**Table 1:** Core Collection evaluation scores for different *K* selected elements

			Group A					Group B			
*K*			6	12	24	48		6	12	24	48
ANE	*data 0*		0.7924	0.7451	0.6851	0.6159		N/A	N/A	N/A	N/A
	*data I*		0.7167	0.6478	0.574	0.4294		0.5283	0.4047	0.3218	0.2279
	*data II*		0.5212	0.3944	0.3262	0.2007		0.7145	0.6496	0.5692	0.4367
	*data III*		0.7338	0.6683	0.5725	0.4322		0.4978	0.4164	0.3126	0.2199
ENE	*data 0*		0.1911	0.2646	0.2574	0.2735		N/A	N/A	N/A	N/A
	*data I*		0.2463	0.2886	0.2961	0.3584		0.4925	0.5548	0.6139	0.7087
	*data II*		0.4204	0.5183	0.574	0.6379		0.2703	0.289	0.3065	0.3519
	*data III*		0.1355	0.2516	0.3109	0.3145		0.4761	0.5329	0.6265	0.6776
E	*data 0*		0.9113	0.8894	0.9059	0.9069		N/A	N/A	N/A	N/A
	*data I*		0.8851	0.888	0.8917	0.8879		0.7604	0.7767	0.7576	0.74
	*data II*		0.7415	0.7671	0.7593	0.7587		0.8905	0.893	0.8815	0.8818
	*data III*		0.9272	0.8957	0.894	0.8915		0.7603	0.7357	0.7395	0.7501
MD	*data 0*		16.5192	4.7198	2.6549	1.7699		N/A	N/A	N/A	N/A
	*data I*		18.3746	9.894	2.1201	0.3534		0	0	0	0
	*data II*		22.2615	13.7809	6.0071	1.4134		22.2615	13.0742	6.0071	1.4134
	*data III*		24.7588	12.8617	1.9293	1.2862		7.1429	0	0	0
VD	*data 0*		27.4336	36.8732	41.0029	46.3127		N/A	N/A	N/A	N/A
	*data I*		33.9223	45.2297	51.2367	53.3569		67.8571	67.8571	57.1429	50
	*data II*		31.8021	38.8693	45.9364	56.1837		30.742	37.4558	44.1696	55.477
	*data III*		35.6913	42.4437	53.6977	54.0193		50	53.5714	67.8571	67.8571
CR	*data 0*		29.7935	46.0177	57.8171	69.9115		N/A	N/A	N/A	N/A
	*data I*		37.1025	55.1237	68.9046	81.9788		71.4286	85.7143	89.2857	100
	*data II*		36.7491	47.7032	62.1908	77.7385		34.2756	45.9364	60.0707	77.0318
	*data III*		41.4791	54.0193	73.6334	81.672		71.4286	85.7143	96.4286	96.4286
VR	*data 0*		27.6275	41.3319	54.425	66.2917		N/A	N/A	N/A	N/A
	*data I*		32.6321	48.6972	63.4782	80.4787		76.7938	86.7248	91.9404	102.2757
	*data II*		34.9972	46.2934	58.241	75.3076		30.9728	43.7712	55.211	74.2049
	*data III*		38.7036	51.9165	70.2887	77.0397		78.3303	93.7485	96.6503	94.5884
CA	*data 0*		64.8968	73.0088	78.9086	84.9558		N/A	N/A	N/A	N/A
	*data I*		68.5512	77.5618	84.4523	90.9894		85.7143	92.8571	94.6429	100
	*data II*		68.3746	73.8516	81.0954	88.8693		67.1378	72.9682	80.0353	88.5159
	*data III*		70.7395	77.0096	86.8167	90.836		85.7143	92.8571	98.2143	98.2143

*ANE*, average distance between each original collection (MC) and nearest core collection (CC) sample; *ENE*, average distance between each CC sample and nearest CC sample; *E*, average distance between CC samples; *MD*, homogeneity test for means; *VD*, homogeneity test for variance; *CR*, coincidence rate; *VR*, variable rate; *CA*, coverage of allele. N/A, not possible to perform diferent-set comparison. With the exception of ANE and MD, higher values suggest better representation. Detailed description of the scoring system is provided in the text. Group A core collections where compared with their original collection dataset; contrarily, when possible, core collections in group B where compared to another equivalent original collection dataset

The discreteness of the 141 accessions used in the CC selection procedures was confirmed by displaying their distribution on the phylogenic dendrogram based on *data 0* presented in Additional file 3 (Online Resource 3). In order to evaluate whether the CC was representative, a phylogenetic dendrogram was constructed based on the genotypic distances among the MCs *data I*. The phylogenetic reconstruction obtained eight groups, which agreed with previously reported groupings [25]. Thus, the selected CCs were identified according to this dendrogram.

**Fig. 5:**
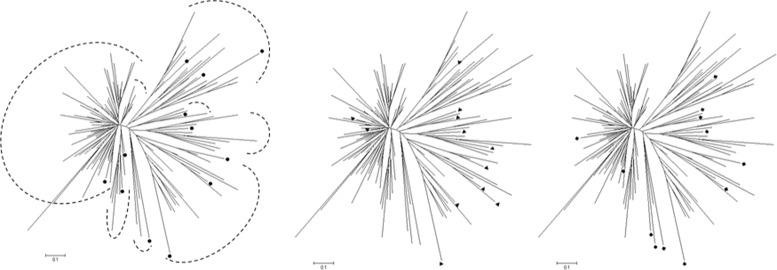
Distribution of the selected CCs (k = 12) from *data I* (*solid circles-left*), *data II* (*solid triangles-center*), and *data III* (*solid squares-right*) based on the dendrogram obtained using 141 foxtail millet individuals. The dashed lines represent groups of clusters

**Fig. 6:**
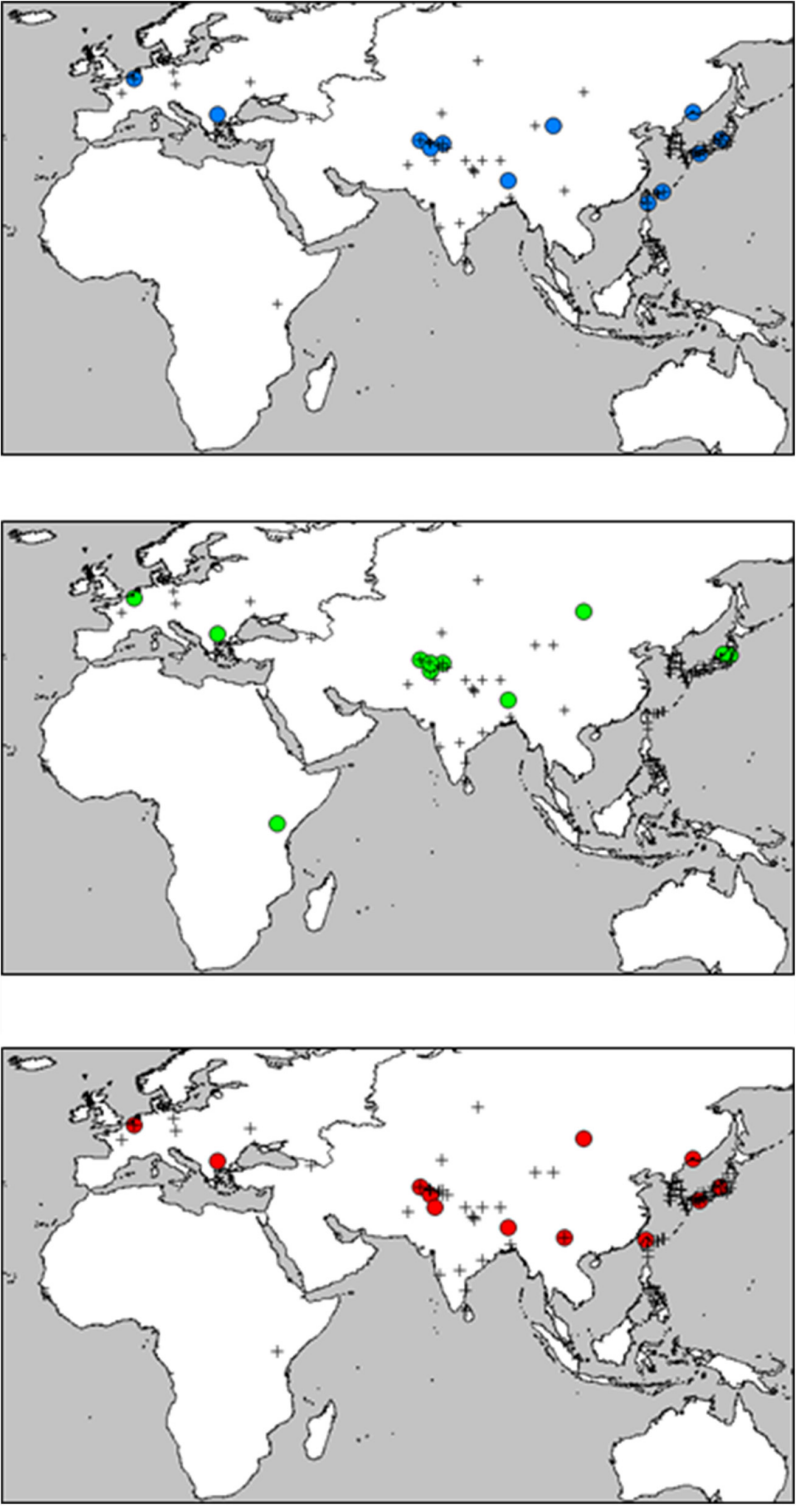
Geographical distribution of *k* = 12 CCs from *data I* (*top*), *data II* (*center*), and *data III* (*bottom*). The colored dots represent the geographical origin of each CC member and the crosses represent the geographical origin of each accession included in the analysis. Maps were generated with Diva-GIS 7.5 http://www.diva-gis.org/ based on GADM v.1.0 http://www.gadm.org/

The distribution pattern of the dendrogram demonstrated that *data I* CC covered the largest number of branches, followed by *data III* and *data II* (Fig. 5). This may be because the tree itself was constructed using *complete data*, which differed from *data I* only in terms of the number of accessions included in each dataset. However, *data II* CC also covered over half of the branches when *K*>12. *Data III* CCs covered as many different branches as *data I* CC (except *K*=48). This suggests that the *data III*-based CCs successfully integrated phenotypic information into the genotypic information, but without altering the distribution in the dendrogram. The geographical distributions of the selected CCs were also displayed on a world map and the results are shown in Fig. 6*Data II* CCs represented the widest geographical distribution range. The CCs include accessions from both the longitudinal and latitudinal range edges, even small *K* CCs (Fig. 6). This clearly indicates that the *data II* CCs represent accessions that are adapted to different environmental conditions. As the number of *K* increased, the distribution range became wider for all the CCs in terms of both the longitude and latitude. Interestingly, several accessions were selected from different datasets. Among these accessions, two were included in 100 % of the CCs irrespective of their original dataset (12 times in 12 CCs), and 5 accessions were present in 66.7 % (8 times out of 12 CCs) to 91.7 % (11 times out of 12 CCs) of the CCs. These accessions may be distantly related to other accessions in terms of both their genetic and phenotypic traits, although the establishment of a phenotype/genotype correlation would require a different approach. Thus, we demonstrated that it is possible to generate adequate CCs using both phenotypic and genotypic information, and it is important to remember that the phenotypic traits employed in this study were selected and mapped arbitrarily only to establish a proof-of-concept with respect to the feasibility of constructing a comprehensive CC based on both genotypic and AT information. Further studies based on the optimization of phenotypic numerical representations are needed to enhance the accurate representation of the available information. We believe that the use of adequate AT mappings and the inclusion of different molecular markers will improve the CC selection process. This methodology could be used to infer ancestry, particularly with low *K* when the algorithm is expected to favor the selection of polyphyletic taxons that would represent unique ancestry for each element in the CC. However, it needs to be taken into consideration that phenotypic traits may affect this expected outcome, and that the algorithm was not designed nor tested for ancestries establishment.

To the best of our knowledge, the present study is the first attempt to combine genotypic and morphological information during CC construction with this approach. It was possible to construct CCs based on both information types using the proposed methodology. As demonstrated by the VS values, the PCA distribution (Figs. 2, 3, and 4), phylogenetic representations (Fig. 5), and geographic distributions (Fig. 6), the phenotypic data provided useful and potentially important information. We believe that genotypic information alone should not be used to generate CCs. In general, morphological information is used to include variation in the CC [11, 18]. Our evaluation of the PCA distribution suggests that both phenotypic and genotypic information have important effects on the selected CCs.

## Conclusions

Our approach was successful in capturing most of the genotypic, phenotypic, and geographical diversity in a small set of individuals. *Data III* CCs were highly representative in terms of both genetic and phenotypic variations. The use of this approach for CC selection may provide beneficial materials in terms of biochemical, morphological, agronomic, and phylogenetic traits, which can be combined with genomic information. The precise definition of phenotypic numerical representations requires further attention, but we believe that combined information CCs will be highly beneficial for breeding improvement, domestication description processes, evolutionary studies, and phenotype/genotype correlation research given the advantages of using adequate CCs for *S. italica* as well as other crops.

## Availability of data and materials

Supporting data and codes are available as additional files.

## Additional files

Additional file 1
**Online Resource 1.** Tables with Genotype/Phenotype information (OR1.rar). ∙FoxtailATMap.cvs : Mapped Foxtail AT where 1 = ’ presence’; 0 = ’abscence’. (corresponds to *data II*). ∙FoxtailGenMap.cvs : Mapped Foxtail Genotype where 1 = ’presence’; 0 = ’abscence’.(corresponds to *data I*). (RAR 18.2 kb)

Additional file 2 ∙ alleleFrecNRet.m ∙ CCvalidation.m ∙ corrI.m ∙ ChooseInitialCenters.m ∙ dcKMeansB.m ∙ DirectkMeans.m ∙ eigdec.m ∙ FFTCompare.m ∙ FFTForSignals.m ∙ findCCinFC.m ∙ randCC.m ∙ pca2.m ∙ README.m ∙ triu.m (RAR 12.4 kb)

Additional file 3
**Online Resource 3.** Phylogenetic Dendrogram of *data 0* and the selected elements of *data II* (dots) in OR3.png. (PNG 56.5 kb)
