# Supplementary figures and images for: Principal components analysis - K-means transposon element based foxtail millet core collection selection method
Source: BMC Genet. 2016 Feb 16;17:42. doi: 10.1186/s12863-016-0343-z (PMC4754896; doi:10.1186/s12863-016-0343-z)

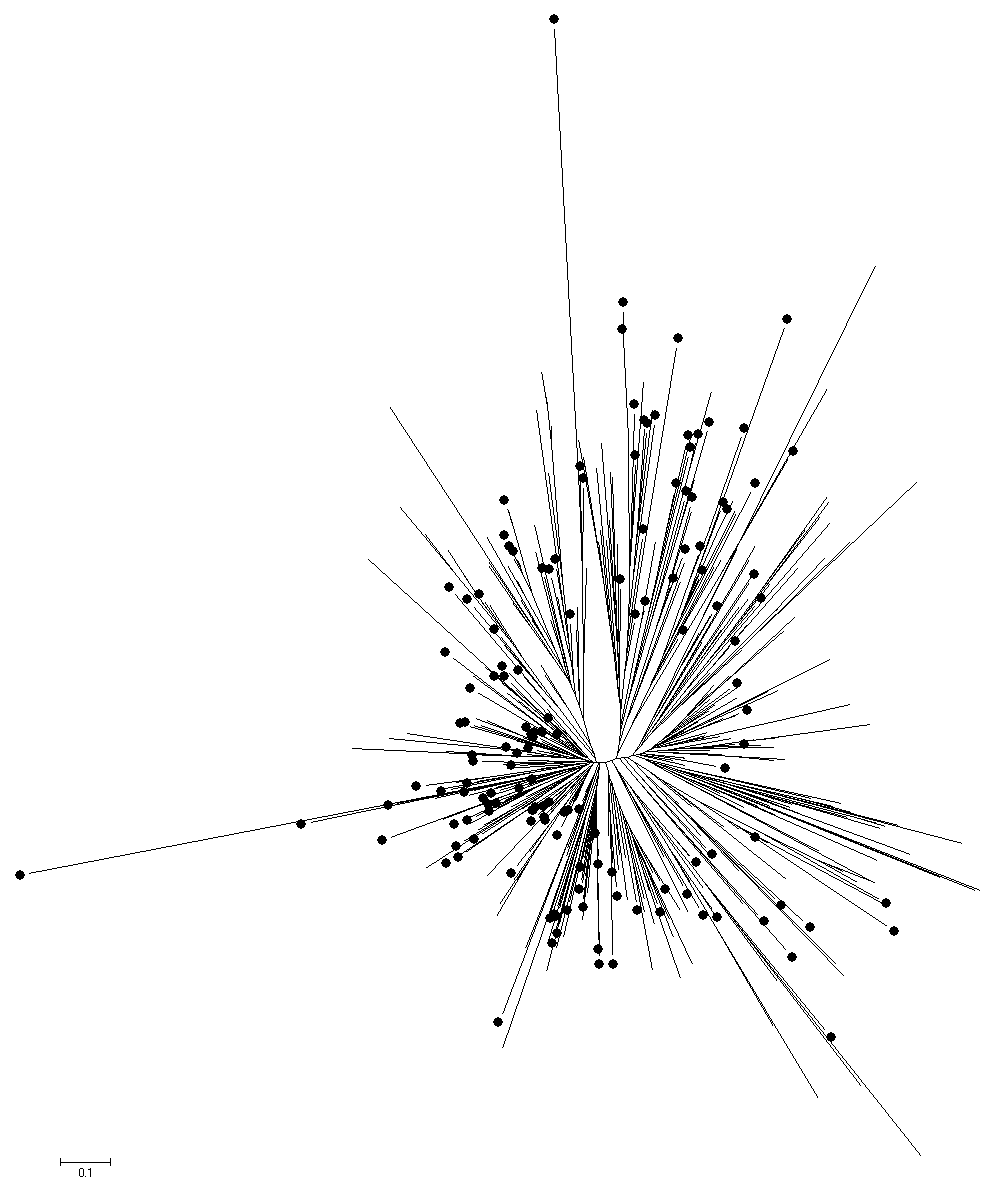

Supplement: Additional file 3 — Online Resource 3. Phylogenetic Dendrogram of data 0 and the selected elements of data II (dots) in OR3.png. (PNG 56.5 kb) [file 12863_2016_343_MOESM3_ESM.png]
